# Influence of Polyurethane Adhesive Formulation on Shear Performance of Maritime Pine Cross-Laminated Timber Under Dry and Moist Exposure Conditions

**DOI:** 10.3390/ma19102030

**Published:** 2026-05-13

**Authors:** Annie Cavalcante, Jorge M. Martins, Margarida Lopes de Almeida, Cláudio Henrique Soares Del Menezzi, Luísa Hora de Carvalho

**Affiliations:** 1 Department of Forest Engineering, Faculty of Technology, University of Brasília, Brasília 70919-970, Brazil; cmenezzi@unb.br; 2 Department of Wood Engineering, Polytechnic Institute of Viseu, 3504-510 Viseu, Portugal; jmmartins@estgv.ipv.pt (J.M.M.); margaridaalmeida@estgv.ipv.pt (M.L.d.A.); lhcarvalho@estgv.ipv.pt (L.H.d.C.); 3 Laboratory for Process Engineering, Environment, Biotechnology and Energy, Faculty of Engineering, University of Porto, 4200-465 Porto, Portugal; 4 Associate Laboratory in Chemical Engineering, Faculty of Engineering, University of Porto, 4200-465 Porto, Portugal

**Keywords:** cross-laminated timber (CLT), polyurethane (PUR), maritime pine, bond-line durability, cure kinetics, shear strength

## Abstract

The construction sector’s drive for sustainability has increased the use of Cross-Laminated Timber (CLT), yet its structural reliability is governed by the integrity of the adhesive bond line. This study evaluates the influence of three one-component polyurethane (PUR) formulations (R1, R2, R3) on the adhesion performance of maritime pine CLT. To isolate adhesive-related effects, lamellas were mechanically classified by modulus of elasticity (MOE) and randomly allocated within stiffness classes. Adhesive characterization through ABES, FTIR, and DSC revealed that R3 exhibited slower cure kinetics (t_0_ = 5482 s) but higher thermal stability. Mechanical testing showed that all formulations developed structurally effective dry bonds with shear strengths exceeding 7.1 MPa, with R3 achieving significantly higher dry shear and interlaminar strength. However, 24 h water immersion caused a catastrophic strength reduction exceeding 95% across all formulations, shifting the failure mode from the wood substrate to the adhesive layer. DSC analysis identified glass transition temperatures between 28 °C and 32 °C, which are consistent with the potential for moisture-induced plasticization near service temperatures. These results indicate that while slower-curing formulations like R3 enhance bond quality in dense softwoods due to improved interphase formation, all evaluated PUR systems showed significant vulnerability to saturated conditions, suggesting that adequate moisture protection is essential for maritime pine CLT applications.

## 1. Introduction

The construction sector is one of the largest contributors to global greenhouse gas emissions, accounting for approximately a quarter of worldwide CO_2_ output [1,2]. In this context, the increased use of wood-based materials has gained attention due to their low embodied energy, renewability, and capacity to sequester carbon during growth [3,4,5,6]. When transformed into long-life construction products, wood can function as a temporary carbon sink, contributing to emission mitigation strategies in the built environment [7,8,9,10,11]. Among timber-based solutions, engineered wood products have emerged as key materials for reducing the environmental footprint of buildings while meeting modern structural requirements.

Global CLT production has grown rapidly, reaching an estimated capacity of 2.8 million cubic meters in 2020, and projections exceed 4 million cubic meters by 2025 [12]. As CLT applications expand, ensuring bond-line durability under service-related conditions becomes increasingly critical to structural reliability and design safety.

Cross-laminated timber (CLT) is a widely adopted engineered wood product composed of an odd number of solid wood layers bonded orthogonally using structural adhesives. This crosswise arrangement enhances dimensional stability, improves load distribution, and provides high in-plane and out-of-plane load-bearing capacity [13,14,15,16]. As a result, CLT has become increasingly adopted in multi-story and mass timber construction. However, despite its favorable mechanical performance, the structural reliability and service life performance of CLT panels are strongly governed by the integrity of the adhesive bond lines, which are subjected to complex stress states and environmental exposure during service [17,18,19,20,21,22].

Adhesives play a crucial role in CLT by transferring stresses between orthogonal layers, enabling composite action under tensile, compressive, and shear loading [22]. Bond-line performance directly influences glue-line shear strength, interlaminar shear resistance, and overall panel durability. Among the adhesive systems currently employed in CLT manufacturing, one-component polyurethane (PUR) adhesives are the most widely adopted systems due to their high cohesive strength, moisture resistance, ambient-temperature curing, absence of formaldehyde, and ease of industrial application [23,24,25,26,27,28].

Despite their widespread use, polyurethane adhesives are not chemically uniform materials. PUR formulations differ substantially in terms of polyol composition, isocyanate content, open time, curing kinetics, and crosslink density, all of which influence polymer network formation and mechanical behavior at the bond line [17,24,28,29,30]. In many CLT studies, however, PUR adhesives are treated as functionally equivalent, with limited attention given to formulation-dependent differences in adhesion performance and durability despite differences in curing kinetics and network architecture [15,17,31,32]. This simplification may obscure critical variations in bond-line behavior, particularly under environmental stressors such as moisture exposure and aging.

The inherent variability of wood as a natural material introduces additional complexity into evaluating adhesive performance in CLT. Key wood properties, such as density, elastic modulus, shrinkage behavior, and moisture response, significantly influence adhesive penetration, stress transfer, and bond interface integrity. Although engineered wood products are designed to redistribute natural defects and minimize the effects of material heterogeneity, uncontrolled variability in wood characteristics can obscure the effects of adhesives in experimental investigations. Therefore, experimental strategies that control stiffness-related variability are essential for isolating formulation-dependent adhesive effects.

This challenge is particularly relevant in CLT production contexts that rely on locally available softwood species with distinct anatomical and mechanical characteristics. In Portugal, manufacturing protocols are largely derived from northern European softwoods, despite the widespread availability of maritime pine (*Pinus pinaster*), a resinous species with comparatively higher density and specific anatomical features that may influence adhesive penetration, curing behavior, and bond-line stability [33,34,35,36]. Evaluating formulation-dependent adhesive performance in maritime pine is therefore essential to ensure reliable CLT production using regionally available resources. The present study was conducted within a collaborative research framework aimed at improving the understanding of adhesive–wood interactions under controlled experimental conditions, with particular emphasis on species-specific characteristics relevant to industrial application.

In this context, the present study aims to investigate the influence of different one-component PUR adhesive formulations on the adhesion performance of maritime pine CLT. It is hypothesized that PUR adhesives with longer open times would form more stable polymer networks and exhibit improved resistance to degradation under moisture exposure.

## 2. Materials and Methods

### 2.1. Materials

Maritime pine (*Pinus pinaster* Ait.) boards were used for the production of CLT panels. The boards were sourced from the local market in Viseu/Portugal, with an average initial moisture content of 12%. The physical properties, including density (basic and oven-dry), moisture content, and shrinkage (tangential, radial, axial, and volumetric), were determined in accordance with NP 615 [37]. The specimens were first saturated in water and subsequently dried at a temperature of 103 ± 2 °C until constant mass was achieved. This procedure was used to determine the density and shrinkage parameters of the specimens. The fibre saturation point (S) was calculated as the ratio between volumetric shrinkage (Rv) and the coefficient of volumetric shrinkage (v).

In order to control variability in stiffness among lamellas, a total of 175 boards were mechanically classified based on modulus of elasticity (MOE). The MOE was determined using a three-point static bending test performed on a universal testing machine under non-destructive conditions. The test span was 420 mm, and the cross-sectional dimensions of the specimens were 100 × 20 mm. The load was applied at a rate of 2.5 mm/min, and deflection was measured at mid-span.

The modulus of elasticity was calculated using Equation (1):

(1) $$MOE=\frac{PL^{3}}{48∆I}$$

where *P* is the applied load (N), *L* is the free span (mm), ∆ is the mid-span deflection (mm), and *I* is moment of inertia (mm^4^).

The distribution of MOE values was used to create a hierarchical classification system, which was then employed to categorize the boards into three stiffness classes with equal numbers of samples per class (n ≈ 58). The classes were designated as follows: Class A, high modulus; Class B, medium modulus; and Class C, low modulus. To minimize variability related to stiffness while maintaining realistic material heterogeneity, lamellas were randomly selected within each stiffness class for CLT panel production.

### 2.2. Adhesive Characterization

#### 2.2.1. ABES Testing

**Figure 1:**
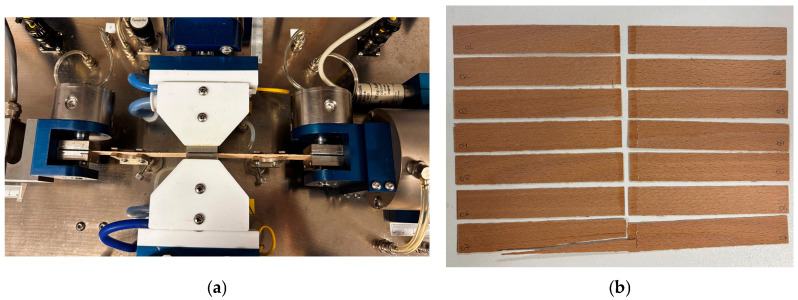
Evaluation of adhesive bonding strength using the ABES system. (**a**) shows wood veneer strips positioned in the ABES device prior to testing; (**b**) presents the tested specimens after shear failure.

An Automated Bonding Evaluation System (ABES; Adhesive Evaluation Systems, Corvallis, OR, USA) was used to evaluate the development of shear strength in wood–adhesive–wood assemblies as a function of pressing time [38,39]. Beech (*Fagus sylvatica* L.) veneer strips with a thickness of 0.7 mm thickness were conditioned at 20 °C and 53% relative humidity for one week prior to testing to ensure uniform moisture content. Beech veneer was selected due to its anatomical uniformity and low extractive content, which minimizes substrate-related variability and enables the intrinsic curing kinetics of the adhesive formulations to be compared under standardized conditions [38]. Veneer strips measuring 117 mm × 20 mm were prepared using a pneumatically driven cutter designed for the ABES system (Figure 1).

Two veneer strips were bonded in the fiber direction with an overlap area of 100 mm^2^. A total of 5 mg of PUR adhesive was applied to each bonding surface. Bonding was performed at ambient laboratory temperature (20 ± 3 °C) under a constant pressure of 2 MPa. PUR adhesives cure through moisture-activated chemical reactions, so no external heating was applied during curing.

Pressing times ranged from 60 s to 4800 s, depending on the formulation of the adhesive. After the stipulated pressing time, specimens were promptly subjected to tensile shear testing within the ABES device at a crosshead speed of 1.0 mm/min. The maximum shear strength was determined. Four replicates were tested for each pressing time and adhesive formulation.

The development of shear strength as a function of time was fitted using the model described in Equation (2).

(2) $$\tau =\frac{\tau_{\infty }}{1+A}\left[A+\tanh\left(\frac{t-t_{0}}{k}\right)\right]\;A=\tanh\left(\frac{t_{0}}{k}\right)$$

where $\tau \left(t\right)$ is the shear strength (MPa) at time t, $\tau_{\infty }$ is the asymptotic maximum shear strength (MPa), $t_{0}$ (s) represents the gel time of the adhesive, and k (s) is the dynamic cure constant associated with cure kinetics.

#### 2.2.2. Fourier-Transform Infrared Spectroscopy (FTIR)

Fourier-transform infrared spectroscopy (FTIR) analysis was performed using a VERTEX 70 FTIR spectrometer (BRUKER, Billerica, MA, USA) equipped with a high sensitivity DLaTGS detector in the mid-infrared region (4000–300 cm^−1^) with a spectral resolution of 1 cm^−1^. Spectra were collected in absorbance mode using a A225/Q PLATINUM ATR diamond crystal module (Bruker, Billerica, MA, USA). Cured adhesive films were prepared from each PUR formulation and analyzed after curing under ambient conditions (24 h at 20 ± 2 °C). For each formulation, spectra were recorded at three different locations on the film and averaged using OPUS 7.5 software to ensure representative characterization.

Peak assignments were made based on characteristic polyurethane functional groups: urethane carbonyl (C=O) at approximately 1700 cm^−1^, N-H stretching at 3300–3500 cm^−1^, hydroxyl groups (OH) at 3200–3600 cm^−1^ (broad absorption), isocyanate (NCO) at approximately 2270 cm^−1^, and C-H stretching at 2920–2960 cm^−1^.

#### 2.2.3. Differential Scanning Calorimetry (DSC)

Thermal characterization of the polyurethane adhesives was performed using a NETZSCH DSC 214 Polyma (NETZSCH-Gerätebau GmbH, Selb, Germany) equipped with a Corona sensor. Approximately 10 mg of adhesive sample was placed in a Concavus aluminum pan with a pierced lid. Measurements were conducted under a nitrogen atmosphere (40 mL/min sample flow, 60 mL/min purge flow).

Dynamic scans were performed from 20 °C to 250 °C at a heating rate of 10 K/min. Temperature and sensitivity calibrations were applied prior to analysis. Thermal transitions were identified from heat flow curves (exo up convention). The glass transition temperature (Tg) was determined from the inflection region of the heat flow curve. Exothermic or endothermic events were analyzed to assess residual curing behavior and thermal stability.

### 2.3. CLT Production

**Figure 2:**
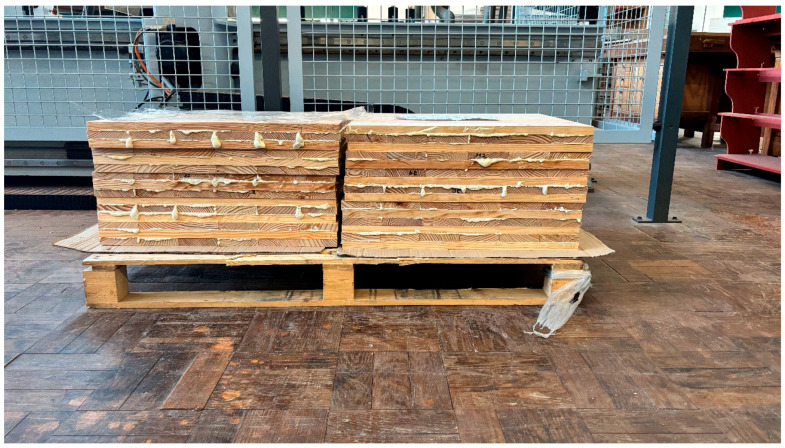
Cross-laminated timber (CLT) panels manufactured using three different types of polyurethane (PUR) adhesives.

Nine three-layer cross-laminated timber (CLT) panels were manufactured from maritime pine (*Pinus pinaster*) lamellas with nominal dimensions of 2 × 10 × 60 cm (thickness × width × length). The final panel dimensions were 60 × 6 × 60 cm (Figure 2).

For each polyurethane formulation (R1, R2, and R3), three panels were produced, totaling nine CLT specimens. To isolate adhesive-related effects from the inherent heterogeneity of the wood, a structured lamella allocation protocol was implemented based on MOE of the boards.

The assembly followed the standard mechanical hierarchy of structural CLT. Lamellas from the higher stiffness classes (Classes A and B) were assigned to the outer longitudinal layers to withstand maximum bending stress, while lower-stiffness lamellas (Class C) were allocated to the central transverse layers. Within the designated layers and stiffness classes, lamellas were randomly assigned to the panel configurations. This systematic approach ensured that the stiffness conditions were homogeneous across all experimental groups. This effectively minimized systematic bias and allowed for a robust assessment of how the specific PUR formulations dictate bond-line performance.

Three one-component polyurethane (1K-PUR) adhesives with distinct open times and viscosities were used (Table 1). Adhesives were applied at a spread rate of 200 g/m^2^ to a single bonding surface. Panels were assembled with orthogonal layer orientation (0°/90°/0°) and pressed under a pressure of approximately 0.3 N/mm^2^ at ambient laboratory conditions (20 ± 2 °C). Pressing times followed the manufacturer’s technical data sheets for each adhesive formulation.

After pressing, panels were conditioned at 20 ± 2 °C and 54% relative humidity for seven days prior to specimen preparation and mechanical testing to ensure complete curing.

### 2.4. CLT Characterization

To assess the effect of different types of PUR adhesives on the adhesion properties of CLT, rolling shear strength and bonding shear resistance were determined, along with wood failure percentages and failure mode analysis.

#### 2.4.1. Bonding Shear Resistance Testing

**Figure 3:**
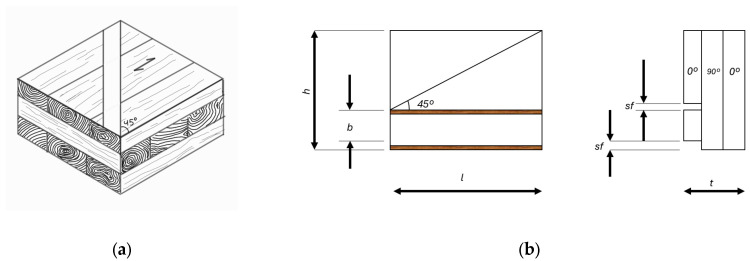

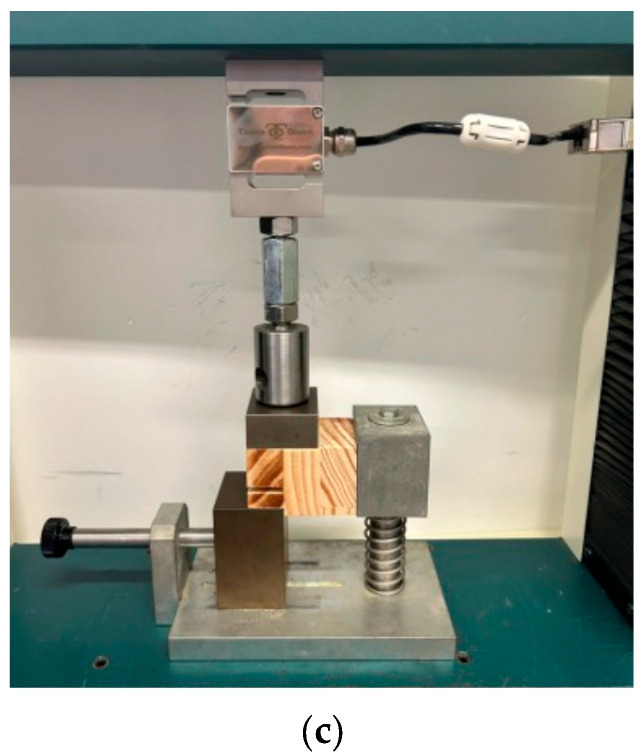
Specimen extraction and bonding shear test configuration according to EN 13354. (**a**) Cutting layout used to obtain shear specimens from the CLT panel; (**b**) schematic representation and dimensions of the shear specimen, where 1 and 2 indicate the grain direction of the outer layers, h is the specimen height (40 mm), b is the shear width (10 mm), sf is the saw-cut width (≥3 mm), and t corresponds to the panel thickness; (**c**) shear testing setup.

Bonding shear resistance was evaluated according to EN 13354 [39]. Test specimens were extracted from the central region of each CLT panel by cutting strips at 45° relative to the panel edges, following the standard cutting layout. Specimens were prepared such that approximately half contained saw cuts in face A and the remaining half in face B to ensure that bond lines in both directions were assessed (Figure 3).

Prior to testing, specimens were conditioned according to the SWP/1 procedure specified in EN 13354. Although SWP/1 corresponds to panels intended for dry service conditions, the standard requires a short water immersion pre-treatment to assess bond-line integrity under moisture exposure. Accordingly, specimens were submerged in water at 20 ± 3 °C for 24 h and tested immediately after removal from water.

Bonding shear resistance (τ, MPa) was calculated by the following Equation (3):

(3) $$\tau =\frac{F_{max}}{b\cdot h}$$

where $F_{max}$ is the maximum load at failure (N), b is the shear width (mm), and h is the specimen height (mm), as defined in EN 13354 (Figure 3).

A total of 10 specimens were tested per panel, corresponding to 90 specimens per adhesive formulation (three panels per formulation). After testing, wood failure percentage (WFP) and failure mode were visually assessed in accordance with EN 13354.

After testing, fractured surfaces were visually inspected and photographed. Wood failure percentage (WFP) was determined in accordance with EN 13354 by estimating the proportion of wood fibers remaining adhered to the bonded interface relative to the total bonded area. Failure modes were classified as follows: <10% (adhesive failure), 10–40% (mixed adhesive–wood failure), 40–90% (predominantly wood failure), and >90% (complete wood failure).

#### 2.4.2. Rolling Shear Testing (Short Beam)

Rolling shear strength was determined using short-span three-point bending tests (L = 5 h), employing a configuration adapted from EN 408 [40] and consistent with the shear-dominated approach proposed by Li (2017) [41]. This span-to-depth ratio promotes shear-dominated failure in the cross layers of cross-laminated timber (CLT) panels.

**Figure 4:**
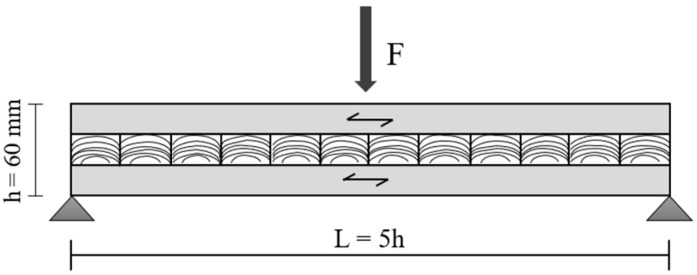
Schematic representation of the short-span three-point bending configuration used to determine rolling shear strength of CLT specimens (span-to-depth ratio L = 5 h). The central layer (90° orientation) represents the cross-laminated layer subjected to rolling shear failure.

Specimens measuring 6 × 6 × 32 cm (width × thickness × length) were extracted from each panel, resulting in a span of 30 cm. Four specimens were obtained per panel, totaling 12 specimens per adhesive formulation. The test setup is illustrated in Figure 4.

Tests were conducted under displacement-controlled loading, with the loading rate adjusted to ensure failure occurred within the time interval specified by EN 408 (300 ± 120 s). Rolling shear strength (τ_(R)_, MPa) was calculated by Equation (4):

(4) $$\tau_{\left(R\right)}=\frac{3F_{max}}{4bh}$$

where $F_{max}$ is the maximum load at failure (N), b is the specimen width (mm), and h  is the panel thickness (mm).

After testing, failure modes were visually evaluated to confirm rolling shear failure in the cross-laminated (90°) middle layer.

### 2.5. Statistical Analysis

In order to verify the effect of the type of adhesive on the adhesive properties of CLT panels, statistical analysis was performed on the shear and short-span bending data at 95% significance level. Welch’s ANOVA was used for analysis of variance, as it does not assume equal variances. The Games–Howell post hoc test was used for pairwise comparisons. Tukey’s HSD test was used to compare means among wood property groups (MOE classes). All statistical analyses were performed using R software (version 4.3.2), with significance set at *p* < 0.05.

## 3. Results

### 3.1. Physical Properties

Table 2 summarizes the physical and dimensional properties of the maritime pine (*Pinus pinaster*) samples.

The mean Ds was 1.180 g/cm^3^ (SD = 0.027), ranging from 1.093 to 1.225 g/cm^3^. The mean D12 and D0 were 0.676 g/cm^3^ and 0.644 g/cm^3^, respectively, both with standard deviations of approximately 0.06 g/cm^3^.

Ra presented a mean value of 0.006%, with values ranging from −0.346% to 0.308%. Rt and Rr averaged 8.443% and 6.606%, respectively, with standard deviations of 1.843% and 1.938%. Rv averaged 15.614%, with values between 7.307% and 21.855%. The coefficient v presented a mean value of 0.543.

The Rt.Rr ratio averaged 1.407, with values ranging from 0.223 to 3.020. The fiber saturation point (S) averaged 29.111%, with a standard deviation of 4.177% and values between 17.146% and 45.802%.

### 3.2. MOE Grouping

**Figure 5:**
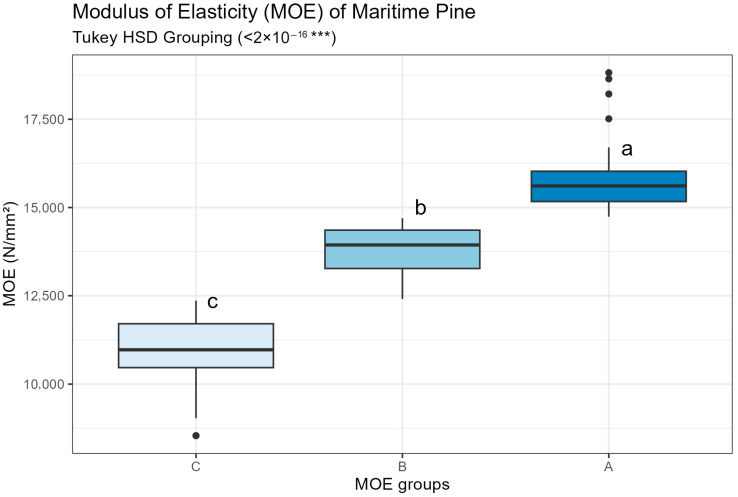
Distribution of modulus of elasticity (MOE) values for maritime pine samples grouped into three stiffness classes (A, B, and C). Different letters (a, b, c) indicate statistically significant differences according to Tukey’s HSD test (*p*-value < 2 × 10^–16^). Note: *** indicates statistical significance at *p* < 0.001.

Figure 5 shows the distribution of the MOE for maritime pine samples categorized into three groups: A, B and C. Statistical analysis using one-way ANOVA differences between the groups (*p*-value < 2 × 10^−16^). Tukey’s HSD test confirmed that all pairwise comparisons were statistically significant, resulting in three distinct groups.

Group A exhibited the highest MOE values, with a median above 15,000 N/mm^2^. Group B presented intermediate values, with a median near 13,500 N/mm^2^. Group C showed the lowest stiffness, with a median below 11,000 N/mm^2^. The interquartile ranges of the groups were separated, and outliers were observed in all classes. The classification procedure effectively produced three statistically distinct stiffness groups for subsequent panel manufacturing.

### 3.3. Adhesive Characterization

#### 3.3.1. ABES

**Figure 6:**
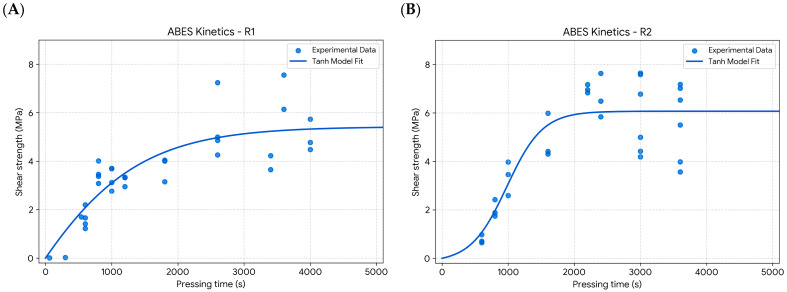

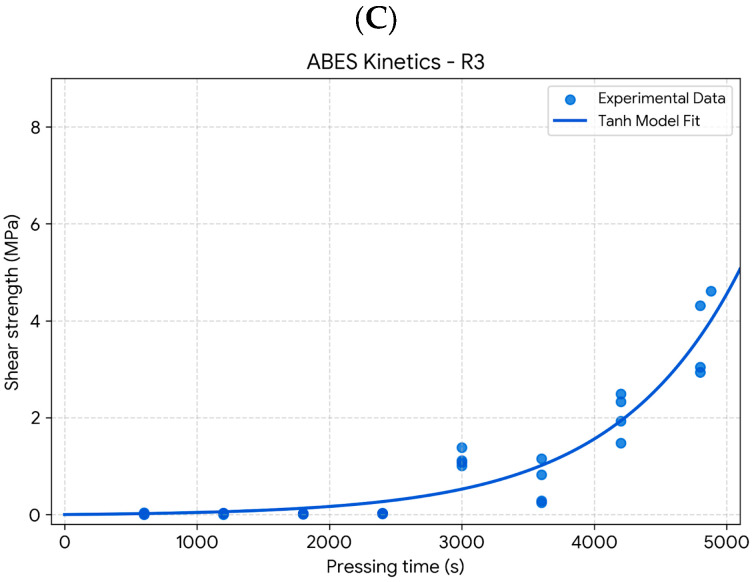
Shear strength development of one-component polyurethane adhesives (R1, R2, and R3) evaluated via the ABES method. Symbols denote experimental shear strength (MPa) relative to pressing time, while solid lines represent the nonlinear regression fits using the sigmoidal cure model. Variations in curve trajectory highlight the distinct, formulation-dependent cure kinetics. **(A)** Resin 1 (R1), **(B)** Resin 2 (R2), and **(C)** Resin 3 (R3).

The development of shear strength as a function of pressing time for the three evaluated one-component polyurethane adhesives (R1, R2, and R3) is presented in Figure 6. The shear strength (τ) was calculated as the ratio between the measured force and the bonded area (100 mm^2^), expressed in MPa.

The experimental data were fitted using the sigmoidal kinetic model described in Equation (2). The fitted kinetic parameters, including asymptotic shear strength (τ∞), transition time (t_0_), cure rate constant (k), and characteristic times to reach 50% and 90% of τ∞ (t50 and t90), are summarized in Table 3.

The curing profiles varied significantly across the three formulations. R1 showed an early onset of strength development with a transition time of t_0_ of 655 s, gradually approaching an asymptotic value of 5.94 MPa. It reached 50% and 90% of this maximum at 1137 s and 2404 s, respectively.

In contrast, R2 featured the most accelerated kinetics. Despite a later transition time (t_0_ = 992 s) compared to R1, its cure rate was markedly higher, achieving 90% of its 6.79 MPa asymptotic strength in just 1721 s.

Conversely, R3 displayed the slowest progression. While its ultimate strength (11.68 MPa) was higher than R1 and R2, its transition time (t_0_ = 5482 s) was substantially delayed. This slower kinetic behavior is evidenced by the 7603 s required to reach the 90% threshold, nearly four times longer than R2.

The model showed satisfactory agreement with experimental data (R^2^: 0.74–0.89). The higher R^2^ for R3 reflects a consistent, predictable gain of strength. In contrast, R1 and R2 exhibited greater data scatter at intermediate time steps, resulting in more modest fit values.

#### 3.3.2. Chemical Structure (FTIR)

**Figure 7:**
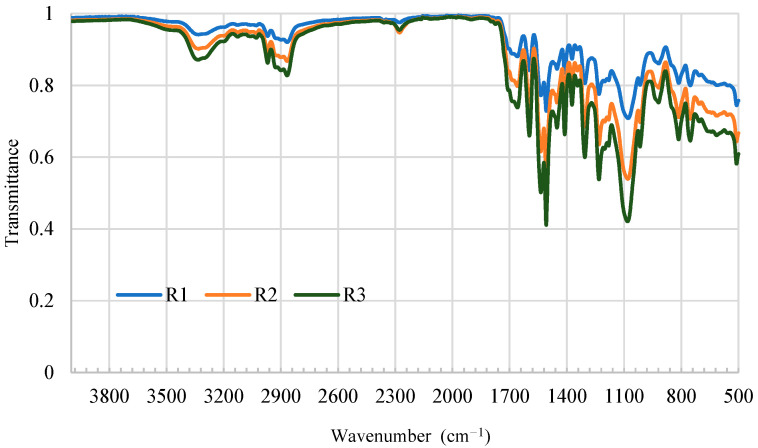
FTIR spectra of fully cured polyurethane adhesive films (R1, R2, and R3) recorded in the mid-infrared region (4000–500 cm^−1^). All formulations exhibit characteristic polyurethane absorption bands, including urethane carbonyl (C=O) stretching near 1700 cm^−1^ and N–H stretching between 3200 and 3500 cm^−1^. The absence of the isocyanate band at approximately 2270 cm^−1^ confirms complete curing. Differences in the fingerprint region (1500–900 cm^−1^) indicate formulation-dependent variations in polymer network organization.

The FTIR spectra of the cured adhesive films are shown in Figure 7. The absence of the characteristic isocyanate (–NCO) absorption band at 2270 cm^−1^ in all samples confirms the complete consumption of reactive groups under the applied curing conditions. In one-component moisture-curing PUR systems, NCO consumption occurs primarily through reaction with atmospheric and wood-bound moisture, forming carbamic acid intermediates that decompose to amine groups and subsequently react with remaining isocyanate groups to form urea linkages, as well as through direct urethane bond formation with hydroxyl groups present in the wood substrate [23,30].

Successful formation of the polyurethane network is further evidenced by the N–H stretching vibrations (3200–3500 cm^−1^) and the urethane carbonyl (C=O) stretching band at approximately 1700 cm^−1^. Symmetric and asymmetric C–H stretching vibrations of the aliphatic backbone were also observed between 2850 and 2950 cm^−1^.

While the primary chemical signatures were consistent, R3 displayed a significantly higher relative intensity in the fingerprint region (1500–900 cm^−1^). Specifically, the absorption band at ~1100 cm^−1^, typically associated with C–O–C stretching vibrations, was more pronounced in R3 than in R1 and R2. Minor variations in the shape of the N–H band also suggest differences in the local chemical environment. These spectral deviations correlate with macroscopic kinetic data, suggesting that the structural variations in R3, likely related to soft-segment concentration or network organization, are responsible for the delayed cure progression observed in the ABES analysis.

#### 3.3.3. Thermal Properties (DSC)

**Figure 8:**
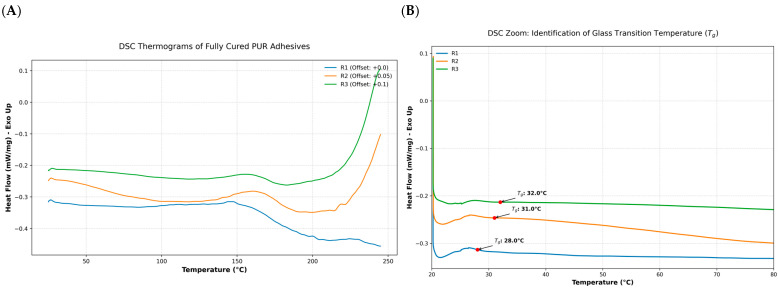
Thermal characterization of the cured PUR adhesives: (**A**) full DSC thermograms (20–250 °C) with vertical offsets; (**B**) zoom-in of the glass transition region (20–80 °C) indicating the identified *Tg* values for each formulation.

Thermal analysis by DSC revealed formulation-dependent differences in the thermal behavior of the cured polyurethane adhesives (Figure 8).

Thermal analysis revealed the phase transition behavior of the PUR adhesives formulations after complete curing. Results in the 20 °C to 250 °C range corroborate that all resins are amorphous polymers with glass transition temperatures (*Tg*) between 25 °C and 35 °C. Resin R1 exhibited a Tg close to 28 °C, whereas R2 and R3 displayed slightly higher values of approximately 31 °C and 32 °C, respectively.

The absence of significant exothermic peaks in the 50 °C region confirms that no intense residual curing occurred. This finding supports the FTIR evidence regarding the complete consumption of isocyanate groups during curing process. However, in the 150 °C region, endothermic peaks were detected for R2 (160.7 °C) and R3 (155.9 °C). These events can be attributed to the dissociation or melting of hard-segment domains within the PUR microstructure, indicating a more defined phase-separated morphology in these formulations [42,43]. In the high-temperature region (>200 °C), formulation R3 exhibited a more stable baseline and a characteristic thermal transition peak at 245.8 °C, indicating a superior thermal stability and a more organized polymer network in comparison to R1 and R2 formulation.

### 3.4. CLT Adhesion Performance

#### 3.4.1. Glue-Line Shear Strength (Dry Conditions)

**Figure 9:**
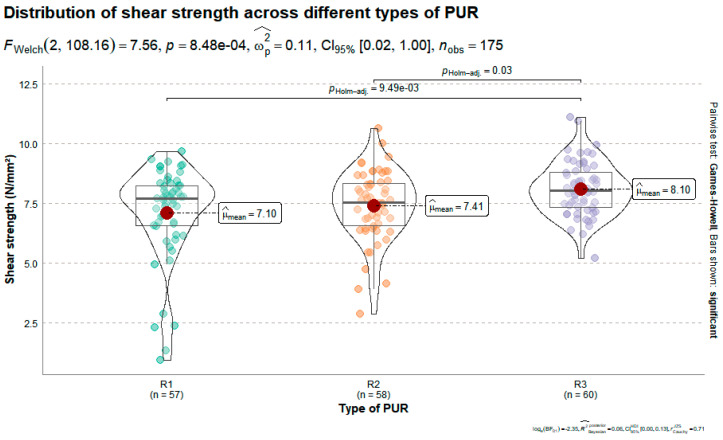
Distribution of shear strength (N/mm^2^) in maritime pine samples bonded with three different polyurethane (PUR) adhesives (R1, R2, and R3). Violin plots display kernel density estimates overlaid with boxplots, individual observations, and mean values (indicated by red dots).

The shear strength of bonded joints using three different types of polyurethane (PUR) adhesives (R1, R2, and R3) was significantly affected by the adhesive formulation, as shown in Figure 9. According to Welch’s ANOVA, there was a statistically significant difference among the groups (*FWelch*_(2, 108_._16)_ *=* 7.56, *p =* 8.48 × 10^−4^), with a moderate effect size (*ω_p_*_2_ *=* 0.11).

The mean shear strength values were 7.10 N/mm^2^ for R1, 7.41 N/mm^2^ for R2, and 8.10 N/mm^2^ for R3. Pairwise comparisons using the Games–Howell post hoc test revealed that the shear strength provided by adhesive R3 was significantly higher than that of both R1 (*pHolm−adj =* 9.49 × 10^−3^) and R2 (*pHolm−adj =* 0.03). No statistically significant difference was found between R1 and R2.

These findings indicate that the R3 formulation achieved the highest bond strength under dry conditions. While these differences are statistically significant, the magnitude of the variation among all three formulations remained moderate. This suggests that all evaluated polyurethane systems were capable of developing structurally effective bonds under dry service conditions.

**Figure 10:**
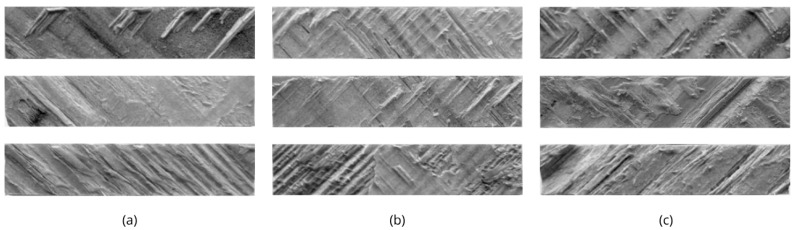
Representative fracture surfaces of glue-line shear specimens after testing (**a**) R1, (**b**) R2, and (**c**) R3 adhesives, illustrating mixed failure modes with varying proportions of wood and adhesive failure. The presence of torn wood fibers adhered to the glue line across all adhesives confirms that failure predominantly occurred within the wood substrate rather than exclusively at the adhesive interface, supporting the wood failure percentages reported in the text.

The analysis of wood failure percentages complemented the shear strength results. Mean wood failure was 48.5% for R1, 57.9% for R2, and 55.0% for R3. All values are within the mixed-to-predominantly wood failure range, indicating that failure generally occurred within the wood rather than exclusively at the adhesive line, and confirming that the three PUR systems developed structurally effective bonds under the tested conditions. The slightly higher wood failure observed for R2 and R3 is consistent with their marginally higher shear strength values and suggests a more efficient stress transfer from the adhesive to the wood substrate (Figure 10).

#### 3.4.2. Wet Shear Strength

**Figure 11:**
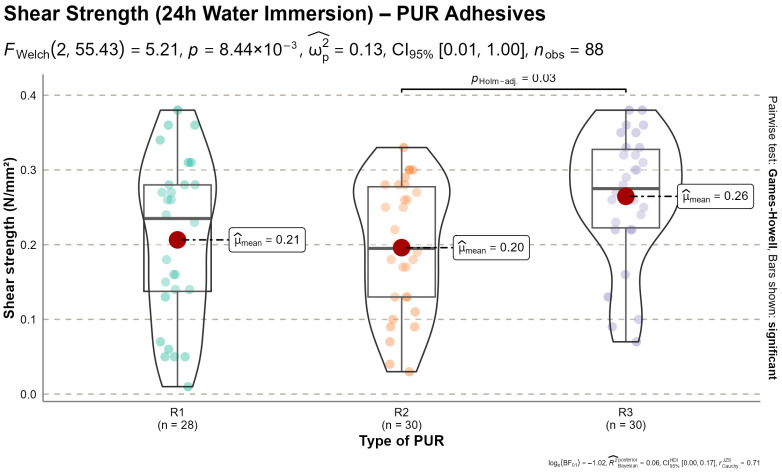
Shear strength (N/mm^2^) of maritime pine samples bonded with three different polyurethane adhesives after 24 h of water immersion. Violin plots display the distribution of values, overlaid with boxplots, individual data points, and mean values (red dots).

Following 24 h of water immersion, shear strength values decreased substantially across all adhesive formulations, indicating pronounced sensitivity of the bonded system to moisture exposure. Welch’s ANOVA confirmed a statistically significant effect of adhesive formulation on wet shear strength (*FWelch*_(2, 55.43)_ = 5.21, *p =* 0.0084)*,* with a moderate effect size (*ωp*^2^ = 0.13), as shown in Figure 11.

The average wet shear strengths were recorded at 0.21 N/mm^2^ for R1, 0.20 N/mm^2^ for R2, and 0.26 N/mm^2^ for R3. While R3 exhibited statistically higher performance compared to R2 (*p* < 0.05), the absolute strength levels for all formulations represented a reduction exceeding 95% relative to the dry-state values (7–8 N/mm^2^).

Moisture exposure also induced a pronounced shift in failure mechanisms. In contrast to the mixed failure modes observed in dry specimens, wet specimens exhibited predominantly adhesive-layer failure. R1 showed 0% wood failure, indicating a complete loss of effective stress transfer to the substrate. Similarly, R2 and R3 exhibited minimal wood failure percentages of 3.1% and 5.0%, respectively. These observations confirm that under saturated conditions, failure occurs primarily within the adhesive bulk or at the interface.

This shift from substrate-dominated to adhesive-dominated failure is consistent with two primary mechanisms commonly reported in the literature: (i) moisture-induced plasticization and softening of the polyurethane network, which is plausible given the proximity of the measured Tg values (28–32 °C) to service temperatures and has been documented for similar PUR adhesive systems [21,24] and (ii) the development of severe hygroscopic stresses. The swelling and distortion of the lamellae induce significant peel and shear stresses at the bond line which, when combined with the reduced cohesive strength of the saturated adhesive, precipitate failure at the interface.

While all formulations exhibited a significant decrease in performance compared to dry conditions, the statistically superior performance of R3 suggests a relatively enhanced resistance to moisture-induced degradation compared to R1 and R2.

#### 3.4.3. Rolling Shear

**Figure 12:**
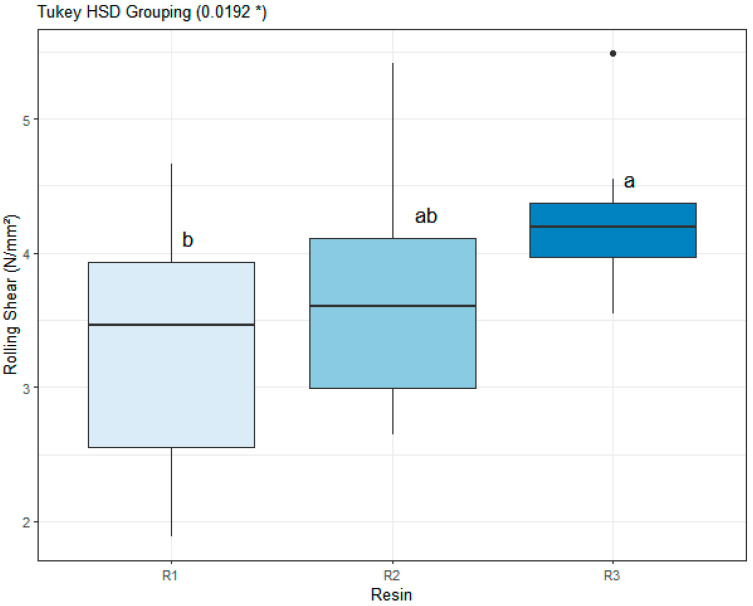
Rolling shear strength (*fr*) of maritime pine (*Pinus pinaster*) CLT panels bonded with polyurethane adhesives. Different letters indicate statistically distinct groups according to Tukey’s HSD test. * indicates statistical significance at *p* < 0.05.

The rolling shear strength (*fr*) of maritime pine CLT panels was significantly influenced by the type of polyurethane (PUR) adhesive (Figure 12). One-way ANOVA revealed a statistically significant effect of adhesive formulation on fr (*p =* 0.0192). Post hoc comparisons using Tukey’s HSD test indicated that panels bonded with R3 exhibited significantly higher rolling shear strength (4.23 MPa; 95% CI: 3.92–4.53) compared to R1 (3.32 MPa; 95% CI: 2.76–3.88). Adhesive R2 showed intermediate values (3.68 MPa; 95% CI: 3.16–4.19) and did not differ significantly from either formulation.

The mean rolling shear strength followed the order R3 > R2 > R1. Although these differences were statistically significant, the magnitude of variation among adhesives remained moderate. This indicates that all three PUR systems developed bond lines capable of effectively transferring shear stress under short-span bending conditions.

**Figure 13:**
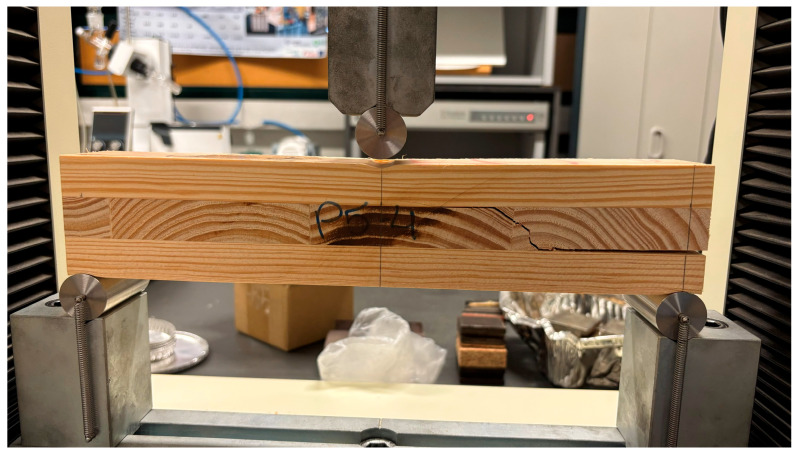
Typical rolling shear failure observed in a short-span bending test of a 3-layer CLT specimen. The horizontal shear crack (visible in the middle transverse layer) propagates along the annual ring lines, leading to separation between the orthogonal layers.

Failure mode analysis revealed that specimens predominantly failed in rolling shear within the transverse lamella rather than at the adhesive interface. The observed horizontal shear cracks propagated along the annual ring orientation of the cross-layer, a behavior consistent with the inherent weakness of wood in shear perpendicular to the grain (Figure 13).

The consistency of rolling shear failure across all formulations indicates that the adhesive bond strength exceeded the shear capacity of the transverse wood layer under the applied loading configuration. Consequently, the performance was limited by the mechanical properties of the wood substrate rather than the interfacial integrity of the adhesive bond.

## 4. Discussion

The present study demonstrates that formulation-dependent characteristics of one-component polyurethane (PUR) adhesives influence cure kinetics and bond-line performance in maritime pine CLT panels. The differences in strength development quantified through ABES parameters such as t_0_ and t_90_, were associated with measurable variations in dry and wet shear strength, indicating that the adhesive formulation affects both processing behavior and structural performance.

The central objective of this research was to determine whether specific PUR formulations could enhance bond-line performance in maritime pine CLT. The results showed that the formulation with the slowest kinetic profile (R3; t_0_ = 5482 s) consistently produced the highest dry shear strength (8.10 MPa) and highest rolling shear strength (4.23 MPa), while the faster-curing R2 (t_90_ = 1721 s) did not yield the highest final mechanical performance.

This behavior indicates that the rapid development of strength in the early stages of curing does not necessarily translate into superior bond integrity in the cured state. The curing speed influences the development of the interphase between the adhesive and the wood, as well as the duration for resin flow before reaching the gel point [30,44]. In the present case, the slower progression of the curing reaction may have allowed greater adhesive mobility prior to network stabilization, which may have favored improved interfacial adaptation in a dense softwood such as maritime pine [45,46].

FTIR analysis confirmed complete curing across all formulations, as evidenced by the disappearance of the isocyanate band at 2270 cm^−1^ and the presence of characteristic urethane carbonyl (~1700 cm^−1^) and N-H stretching bands (3200–3500 cm^−1^). Minor variations observed in the fingerprint region indicate formulation-dependent differences in polymer network organization [18,47]. In one-component PUR adhesives, the balance between soft and hard segments influences flexibility, cure development, and environmental resistance, and polyether-based backbones may be particularly sensitive to moisture exposure [23,24,30,48]. The spectral profile of R3, particularly in the 1000–1200 cm^−1^ region, where C–O–C stretching vibrations are commonly observed, suggests differences in network organization relative to R1 and R2; however, quantitative analyses such as peak deconvolution or band ratio evaluation would be required to support more detailed molecular-level interpretation.

The DSC results further support the existence of formulation-dependent differences in the cured polymer networks, as suggested by the FTIR results. All formulations exhibited thermal transitions consistent with the predominantly amorphous behavior of the polymer, but R2 and R3 exhibited distinct thermal events in the intermediate temperature range. In addition, R3 showed an additional high-temperature event at 245.8 °C, indicating a different thermal response when compared to R1 and R2. Although the results do not allow any direct conclusions about crosslink density, phase separation, or thermal stability classification, they reinforce that the formulations do not respond in the same way to heating and therefore not only differ in their curing kinetics but also in their post-curing thermal behavior. Similar behavior has been reported for PUR adhesives, in which DSC analysis showed no evidence of further polymerization during heating and slightly variations in glass transition temperature under different curing conditions [49]. Those authors also showed that mechanical performance may vary even when Tg remains relatively unchanged, indicating that thermal transitions alone do not fully explain adhesive strength development or environmental sensitivity. In the present study, this is particularly relevant because the cured formulations showed distinct mechanical behavior under dry and wet conditions despite relatively similar thermal transitions.

Under dry conditions, all PUR formulations developed structurally effective bond lines, as indicated by glue-line shear strengths above 7 MPa and a predominantly wood-related failure mode. These results are consistent with previous studies reporting satisfactory dry-state performance of PUR-bonded CLT elements [34,50]. The superior dry shear strength obtained with R3 suggests that adhesive formulation affected the efficiency of stress transfer at the bond line, even though all systems could produce mechanically competent joints under laboratory conditions. Because wood failure percentages remained within the mixed-to-predominantly wood-failure range, the dry-state results indicate that the bonded interface was not the primary weak point in the panel assembly.

Moisture exposure, however, severely altered the bond performance. Following 24 h of water immersion, shear strength values decreased by more than 95% relative to the dry condition, and the failure mode shifted from wood-dominated to predominantly adhesive-layer failure. Similar moisture-induced reductions in bond performance have been reported for structural wood adhesives and one-component PUR systems exposed to hydrothermal or high-humidity conditions, where internal stresses generated by swelling and shrinkage during wetting cycles can compromise bond integrity [21,24,34,51]. Previous studies found that even when shear strength requirements are met, delamination performance in maritime pine CLT may not always comply with standards, highlighting its sensitivity to moisture-induced stresses [34]. Therefore, the collapse observed under wet conditions in the present study is not only a consequence of adhesive plasticization but also a result of species-specific anatomical and chemical characteristics.

It should be noted that the procedure employed in this study is a standardized quality control protocol rather than a simulation of service-level humidity exposure. The catastrophic strength reductions observed indicate sensitivity to saturated conditions but should not be directly extrapolated to in-service wet performance.

In addition, the dimensional instability of maritime pine, reflected by the volumetric shrinkage values measured in this study, may contribute to stress development at the bond line during moisture uptake and swelling. Although R3 showed statistically higher wet shear strength than R2, the residual strength levels remained extremely low for all formulations, indicating pronounced sensitivity to saturated conditions. These findings suggest that the evaluated adhesive systems are better suited for dry service applications. They also indicate that adequate moisture protection is essential when designing maritime pine cross-laminated timber elements.

Furthermore, the combination of high density and elevated extractives content in maritime pine reinforces the need for surface preparation techniques. Previous studies have shown that the application of primers or surface activation treatments can significantly improve the bonding performance of PUR adhesives in resin-rich softwoods by enhancing wettability and reducing the influence of extractive surface barriers [33,50]. That said, the poor performance observed under saturated conditions may not only reflect intrinsic limitations of the adhesive systems but also the absence of surface treatments capable of mitigating species-specific adhesion constraints.

The predominance of rolling shear failure within the transverse lamella indicates that, under the tested loading configuration, the bond lines were sufficient to transfer shear loads to the wood substrate without adhesive failure. Nevertheless, the statistically significant differences in rolling shear strength observed among formulations suggest that the adhesive indirectly influenced the mechanical response of the panel. Formulation-dependent properties such as adhesive elastic modulus and bond-line thickness may alter the distribution of interlayer shear stresses across the orthogonal layers, thereby affecting the apparent rolling shear strength even when failure is ultimately governed by the wood substrate [51,52].

This behavior is consistent with established CLT mechanics, in which transverse shear properties govern failure under short-span out-of-plane loading [13,40]. Likewise, previous studies on maritime pine cross-layered elements have emphasized the importance of using bond quality assessment configurations that minimize rolling shear interference so that the measured response more closely reflects bond-line performance [34]. In the present study, the occurrence of failure in the middle lamella reinforces that panel response was governed primarily by the orthogonal layer arrangement and the inherent weakness of wood in shear perpendicular to the grain, rather than by premature adhesive failure. Therefore, it can be concluded that the magnitude of rolling shear strength was influenced by the adhesive formulation, without altering the wood-controlled nature of the failure mode.

## 5. Conclusions

The present study demonstrated that the formulation of one-component polyurethane adhesives exerts a significant influence on the adhesion performance of maritime pine CLT panels. Even though all formulations achieved complete curing, they exhibited differences in cure kinetics, thermal response, and mechanical behavior. The adhesive with the slowest kinetic profile (R3) exhibited the highest dry glue-line shear strength and rolling shear strength, suggesting that the delayed cure development did not compromise the ultimate bond performance. However, all formulations exhibited a significant loss in strength following water immersion, indicating a notable sensitivity to moisture exposure. It should be noted, however, that the SWP/1 procedure represents a standardized quality control protocol rather than a simulation of in-service humidity exposure, and cyclic aging tests would be required before definitive service class recommendations can be made. Furthermore, they underscore the paramount role of moisture in determining the reliability of bond lines in maritime pine CLT.

## Figures and Tables

**Table 1:** Technical specifications of PUR adhesives used for wood applications. The table summarizes key properties of the adhesives, including manufacturer information, open time, pressing time, and viscosity values as reported in the respective technical data sheets.

Resin	Manufacturer	Open Time (min)	Pressing Time	Viscosity
R1	Company A	7	30 min	6000 mPa·s (Brookfield, sp 4, 20 rpm)
R2	Company B	10	2.5× open time	20,000–32,000 mPa·s (Brookfield, sp 6, 20 rpm)
R3	Company B	30	2.5× open time	24,000 mPa·s (Brookfield, sp 6, 20 rpm)

**Table 2:** Descriptive statistics (mean, median, standard deviation, quartiles, minimum, and maximum) for density and shrinkage properties of Maritime pine (*Pinus pinaster* Sol. ex Aiton).

	Ds	D_12_	D_0_	Ra	Rt	Rr	Rv	v	Rt.Rr	S
Mean	1.180	0.676	0.644	0.006	8.443	6.606	15.614	0.543	1.407	29.111
Median	1.185	0.671	0.643	0.002	8.606	6.564	16.063	0.559	1.335	28.437
Stad. Dev	0.027	0.063	0.061	0.063	1.843	1.938	2.914	0.111	0.496	4.177
Minimum	1.093	0.513	0.482	−0.346	2.575	1.775	7.307	0.278	0.223	17.146
Q25	1.169	0.637	0.603	−0.009	7.547	5.931	14.275	0.496	1.145	27.094
Q75	1.198	0.719	0.681	0.020	9.366	7.556	17.323	0.604	1.586	29.926
Maximum	1.225	0.813	0.762	0.308	12.541	11.525	21.855	0.916	3.020	45.802

Note: Ds = Density (g/cm^3^); D_12_ = Density at 12% moisture content (g/cm^3^); D_0_ = Oven-dry density (g/cm^3^); Ra = Shrinkage (%) from green to oven-dry moisture content (axial); Rt = Shrinkage (%) from green to oven-dry moisture content (tangential); Rr = Shrinkage (%) from green to oven-dry moisture content (radial); Rv = Volumetric shrinkage (%); v = Coefficient of volumetric shrinkage (%); S = Fiber saturation point (%).

**Table 3:** Kinetic parameters obtained from nonlinear regression of ABES shear strength development using the sigmoidal cure model.

Resin	τ∞ (MPa)	t_0_ (s)	k (s)	t50 (s)	t90 (s)	R^2^	RMSE (MPa)
R1	5.94	655.49	880.28	1137.42	2404.14	0.748	1.031
R2	6.79	992.06	494.06	1116.14	1721.75	0.763	1.205
R3	11.68 *	5482.22	1748.01	5848.51	7603.25	0.895	0.422

Note: τ∞ represents the asymptotic shear strength (MPa), t_0_ the transition time (s), k the cure rate constant (s), and t50 and t90 the times required to reach 50% and 90% of τ∞, respectively. RMSE indicates the root mean square error of the fit and R^2^ the coefficient of determination. * The value of τ∞ in R3 is a mathematical extrapolation.

## Data Availability

The original contributions presented in this study are included in the article. Further inquiries can be directed to the corresponding author.
